# Impact of on-site compared to off-site testing for severe acute respiratory coronavirus virus 2 (SARS-CoV-2) on duration of isolation and resource utilization

**DOI:** 10.1017/ice.2020.433

**Published:** 2020-08-24

**Authors:** Andrew T. Roberts, Gabriella Wong, Despina Kotsanas, Michelle J. Francis, Rhonda L. Stuart, Maryza Graham, Benjamin A. Rogers

**Affiliations:** 1 Monash Infectious Diseases, Monash Health, Clayton, Victoria, Australia; 2 Department of Microbiology, Monash Pathology, Monash Health, Clayton, Victoria, Australia; 3 Infection Prevention & Epidemiology, Monash Health, Clayton, Victoria, Australia; 4 Monash University School of Clinical Sciences, Monash Health, Clayton, Victoria, Australia; 5 Microbiological Diagnostic Unit Public Health Laboratory, The Peter Doherty Institute for Infection and Immunity, The University of Melbourne, Melbourne, Victoria, Australia

## Abstract

Rapid detection and isolation of coronavirus disease 2019 (COVID-19) patients is the only means of reducing hospital transmission. We describe the impact of implementation of on-site severe acute respiratory coronavirus virus 2 (SARS-CoV-2) reverse-transcription polymerase chain reaction (RT-PCR) testing on reducing turnaround time, isolation duration, pathology test ordering, and antibiotic use in patients who do not have COVID-19.

Early detection and isolation are key measures in controlling transmission of severe acute respiratory coronavirus virus 2 (SARS-CoV-2).^1^ Reduction in testing turnaround times (TATs) and duration that patients who test negative for SARS-CoV-2 spend in isolation may help relieve healthcare burdens associated with preventing SARS-CoV-2 transmission.^2–4^

On January 24, 2020, the first confirmed case of coronavirus disease 2019 (COVID-19) in Australia was admitted to our health network, Monash Health.^5^ Our hospital at the beginning of the outbreak relied on the regional viral reference laboratory for SARS-CoV-2 testing.^6^ As the pandemic developed, testing for SARS-CoV-2 was performed on site in an attempt to reduce result TAT. Herein we report our experiences of how on-site compared to off-site SARS-CoV-2 testing impacted the duration of time patients who did not have a diagnosis of COVID-19 spent in isolation precautions and subsequent healthcare outcomes.

## Methods

We examined SARS-CoV-2 reverse-transcription polymerase chain reaction (RT-PCR) results on respiratory specimens (nasopharyngeal swabs, endotracheal aspirates, sputum) conducted during March 1–27, 2020, on hospitalized patients at Monash Health, a tertiary metropolitan healthcare network of 4 acute-care hospitals that serves a catchment area of 1.3 million people within Victoria, Australia.

We included all hospitalized patients on isolation precautions as determined by SARS-CoV-2 RT-PCR testing conducted during the study period. Indications for testing were adopted from the national guidelines at that time.^7^ Patients were excluded if the SARS-CoV-2 RT-PCR was positive or if results of testing did not affect isolation duration (ie, they had other indications to remain in precautions). The study period captured 3 phases of SARS-CoV-2 testing. In phase 1 (off-site testing, March 1–14), all tests were sent to the reference laboratory. In phase 2 (early on-site testing, March 15–22), on-site testing began; however, some samples were still sent to the reference laboratory due to reagent shortages or for assay validation. Only tests performed on-site in this period were included. In phase 3 (established on-site testing, March 23–27), all tests were analyzed on site.

Off-site tests were transported ~30 km to the Victorian Infectious Diseases Reference Laboratory via 2 scheduled courier runs per day. The assay used has been described previously.^6^ On-site analysis occurred at the main hospital’s laboratory and was performed with AusDiagnostics (Mascot, Australia) 8-well coronavirus assays (cat. no. 20081) or AusDiagnostics Respiratory Pathogens 12-well assays (cat. no. 80618). Samples taken at peripheral sites in our network were transported to the central laboratory hourly.

Isolation time was defined as the time from when patients were recorded to be in isolation to the time that isolation was recorded as ceased. Result TAT was defined as the interval between test collection and result validation by microbiology staff. Time to notification was defined as the interval between test collection and when a clinician recorded notification of the result.

Categorical variables were compared using the χ^2^ test. Continuous variables were compared with the Kruskal-Wallis rank test. *P* < .05 was considered statistically significant. Statistical analyses were performed using Stata version 13 software (StataCorp, College Station, TX).

## Results

Baseline demographics and clinical characteristics of the tested population are reported in Supplementary Table 1 (online). In total, 242 RT-PCR tests from 220 patients resulted in 224 isolation periods. Of tests linked to isolation periods, 55 (25%) tests were sent off site, 51 (23%) were early in on-site testing, and 118 (53%) took place after on-site testing was established. Groups were matched for age and sex. Higher rates of ICU admission (*P* < .001) and mechanical ventilation (*P* = .024) were observed in the off-site group.

Characteristics of testing are reported in Table 1. The median reported TAT of the established on-site RT-PCR test was lower than that for both early on-site and off-site testing: 16.1 versus 24.3 versus 70.4 hours (*P* < .001). The median time to notification of established on-site RT-PCR results was lower than that for both early on-site and off-site RT-PCR results: 19.7 versus 37.6 versus 47.3 hours (*P* < .001). In the early group who had on-stie tests, there were lower rates of fever being a reason for testing compared to the other two2 cohorts (*P* = .003). To assess the change in TAT within each study phase, data were plotted, and a linear regression line was fitted (Supplementary Fig. 1 online). The TAT over the duration of the off-site phase slowed, which was not seen in the on-site phases.

**Table 1.** Characteristics of Performed SARS-CoV-2 RT-PCR Tests

Characteristic	Off-Site RT-PCR Tests (n=65)	Early On-Site RT-PCR Tests (n=54)	Established On-Site PCR Tests (n=123)	*P* Value
Median report TAT, h (IQR)	70.4 (46.5–111.5)	24.3 (19.4–44.2)	16.1 (14.4–19.1)	<.001
Median time to notification to clinician, h (IQR)*	47.3 (41.5–77.8)	37.6 (25.4–47.1)	19.7 (16.3–23.8)	<.001
**Location of testing**				.274
ED or outpatient clinic, no. (%)	48 (74)	46 (85)	100 (81)	
Hospital ward, no. (%)	17 (26)	8 (15)	23 (19)	
**Specimen type**				.120
Nasopharyngeal swab, no. (%)	58 (89)	52 (96)	119 (97)	
Lower airway (endotracheal aspirate or sputum), no. (%)	7 (11)	2 (4)	4 (3)	
**Indications for testing**				
Respiratory, no. (%)	58 (89)	49 (91)	98 (80)	.084
Fever, no. (%)	46 (71)	22 (41)	77 (63)	.003
Contact of a known case, no. (%)	10 (15)	7 (6)	8 (15)	.054
Repeat testing, no. (%)	10 (15)	4 (7)	10 (8)	.260

Note. RT-PCR, reverse-transcriptase polymerase chain reaction; TAT, turnaround time; IQR, interquartile range; ED, emergency department.

*Data was not available for 8 patients.

Isolation-based outcomes are presented in Table 2. The median isolation time for patients who had tests during the established on-site RT-PCR phase (21.9 hours; IQR, 18.6–27.8) was lower than the isolation time for patients who had testing during the early on-site phase (39.8 hours; IQR, 26.9–47.7) as well as for patients tested during the off-site phase (66.8 hours; IQR, 47.1–97.0) (P < .001). The median number of routine blood tests for patients whose tests were processed onsite was lower than for patients whose tests were sent off site (P < .001). Antibiotic use was lower in the established on-site group than in the off-site and early on-site cohorts (P = .001). Oseltamivir use was higher in the established on-site group than in the off-site and early on-site cohorts (P < .001).

**Table 2.** Time Spent in Isolation and Isolation-Based Outcomes

Variable	Off-Site RT-PCR Tests (n=55)	Early On-Site RT-PCR Tests (n=51)	Established On-Site RT-PCR tests (n=118)	*P* Value
Median time to cessation of isolation, h (IQR)	66.8 (47.1–97.0)	39.8 (26.9–47.7)	21.9 (18.6–27.8)	<.001
Median total number of routine blood tests performed during isolation, no. (IQR)	7 (4–11)	4 (3–7)	4 (2–7)	<.001
Median rate of routine blood tests^a^ performed per day in isolation, no. (IQR)	2.5 (1.5–4.1)	2.9 (1.7–4.6)	4.1 (2.0–6.3)	.006
Median total number of microbiological investigations performed during isolation, no. (IQR)	1 (0–3)	1 (0–2)	1 (0–2)	.085
Median rate of microbiological investigations^b^ performed per day in isolation, no. (IQR)	0.5 (0–1.0)	0.5 (0–1.4)	0.7 (0–1.8)	.6802
Median total number of diagnostic imaging investigations performed during isolation, no. (IQR)	1 (0–1)	1 (0–1)	1 (0–1)	.193
Median rate of diagnostic imaging^c^ investigations performed per day in isolation, no. (IQR)	0.3 (0–0.6)	0.9 (0–1.3)	0.5 (0–1.0)	.009
Received antibiotics^d^ in isolation, no. (%)	48 (87)	45 (88)	79 (67)	.001
Received oseltamivir in isolation, no. (%)	33 (60)	40 (78)	108 (92)	<.001

Note. RT-PCR, reverse-transcriptase polymerase chain reaction; IQR, interquartile range.

a Each full blood examination, urea, electrolytes, creatinine, liver function tests and C-reactive protein performed on a patient was considered a routine blood test.

b Each sputum culture, blood culture, and urine culture performed on a patient was considered a microbiological investigation

c All diagnostic imaging investigations were included.

d Antibiotics given for treatment of the acute presentation (ie, long-term antibiotics prophylaxis, if present, were not included).

## Discussion

We investigated the impact of on-site SARS-CoV-2 RT-PCR testing compared to off-site testing on the time patients who did not have a diagnosis of COVID-19 spent in isolation precautions. We found that the use of on-site RT-PCR testing was associated with reduced isolation time compared to off-site testing for patients who tested negative for SARS-CoV-2.

Although this result is unsurprising, we believe is important. To our knowledge, no other study has assessed the impact of SARS-CoV-2 RT-PCR TATs on healthcare resource utilization. Our results suggest that by performing testing on-site, TATs are reduced and with it, the associated costs of isolation requirements likely also decrease. Reduced respiratory virus RT-PCR TATs themselves have been demonstrated to reduce length of stay and healthcare costs.^8^ In a 2015 Spanish study, rapid influenza RT-PCR testing of hospitalized patients decreased isolation time by 23.7 hours and reduced hospitalization costs by US$70 per patient.^9^

Early on-site testing was also associated with reduced isolation times compared to off-site testing. We originally hypothesized that TATs during the early phase of on-site RT-PCR implementation would be longer than established off-site testing due to the labor required for implementing new testing protocols in a pandemic setting. Interestingly, we discovered that TATs for samples sent to the reference laboratory slowed over the 14-day period. We surmise that through this time, the testing load on the reference laboratory increased precipitously, highlighting one challenge of operating in pandemic settings.

We observed a positive correlation between number of routine blood tests and isolation duration. This was not observed for other secondary investigations. Clinicians may have felt inclined to recheck routine blood tests while waiting for RT-PCR results. Furthermore, this finding may suggest that longer isolation periods serve no additional benefit to patients for number of impactful investigations received and may only add additional resource costs.

By observing distinct time periods in testing protocol at our health network, we attempted to provide insight into our testing practices as it unfolded throughout the pandemic. As such, generalization of our results is limited by our specific experiences. The study population also varied over the study period. Initial guidelines recommended testing patients with severe pneumonia before the indications were broadened. This led to disproportionate testing being associated with ICU admission, mechanical ventilation, and antibiotic use in phase 1. We were also unable to measure the impact of our findings on patients with COVID-19.

SARS-CoV-2 RT-PCR tests on site, both in early and established iterations, were associated with reduced TATs and subsequent isolation times. With the COVID-19 pandemic ongoing, our findings support the benefits of faster establishment of on-site SARS-CoV-2 RT-PCR testing in centers capable of doing so.

## Supplementary material

For supplementary material accompanying this paper visit http://dx.doi.org/10.1017/ice.2020.433. click here to view supplementary material
