# Supplementary material for: Impact of on-site compared to off-site testing for severe acute respiratory coronavirus virus 2 (SARS-CoV-2) on duration of isolation and resource utilization
Source: Infect Control Hosp Epidemiol. 2020 Aug 24:1–3. doi: 10.1017/ice.2020.433 (PMC8245332; doi:10.1017/ice.2020.433)
Supplement: Supplementary file 1 [file icesup.zip › S0899823X2000433Xsup002.docx]

### Supplementary Table 1 – Baseline demographic and clinical characteristics of testing in the study population*

|  | Send-away RT-PCRs  (n=55) | Early on-site RT-PCRs  (n= 49) | Established on-site PCRs  (n=116) | P-value |
| --- | --- | --- | --- | --- |
| Age – yr, median (IQR) | 63.6 (43-74) | 58 (36-73) | 65 (42-80) | 0.419 |
| Male sex – no. (%) | 33 (60) | 25 (51) | 61(53) | 0.587 |
| Overseas Travel^1^ – no. (%) | 15 (27) | 5 (4) | 2 (4) | <0.001 |
| ICU^2^ – no. (%) | 17 (31) | 11 (22) | 10 (9) | <0.001 |
| Mechanical ventilation^2^ – no. (%) | 5 (9) | 5 (10) | 2 (2) | 0.024 |
| **Treating team**  General Medicine – no. (%)  Respiratory – no. (%)  Infectious Diseases – no. (%)  Other medical unit – no. (%)  Surgical unit – no. (%)  Paediatric unit – no. (%)  Psychiatry – no. (%) | 35 (63.6)  4 (7.3)  4 (7.3)  5 (9.1)  2 (3.6)  5 (9.1)  0 | 25 (51.0)  7 (14.3)  4 (8.2)  9 (18.4)  0  4 (8.2)  0 | 53 (45.7)  6 (5.2)  1 (0.9)  27 (23.3)  13 (11.2)  13 (11.2)  3 (2.6) |  |
| **Final diagnoses**  Other respiratory virus – no. (%)  Pneumonia – no. (%)  Exacerbation of airways disease – no. (%)  Urinary tract infection – no. (%)  Cardiac disease – no. (%)  Fever of unknown origin – no. (%)  Other known infection – no. (%)  Unknown or other diagnosis – no. (%) | 15 (27.3)  21 (38.2)  6 (10.9)  2 (3.6)  2 (3.6)  2 (3.6)  5 (9.1)  2 (3.6) | 7 (14.3)  18 (36.7)  5 (10.2)  1 (2.0)  3 (6.1)  3 (6.1)  2 (4.1)  10 (20.4) | 25 (21.6)  25 (21.6)  12 (10.3)  4 (3.4)  7 (6.0)  20 (17.2)  10 (8.6)  13 (11.2) |  |
| *Patients with multiple tests were included in the group of their first recorded test for demographic purposes. Differences in sample numbers occurred as 21 patients had two or more RT-PCRs performed in the study period (two RT-PCRs n=20, three RT-PCRs n=1); and four patients had two separate isolation periods  ^1^Overseas travel taken in the last 14 days prior to admission  ^2^ICU admission or mechanical ventilation at any time during the index admission. | | | | |
