# Supplementary figures and images for: Impact of on-site compared to off-site testing for severe acute respiratory coronavirus virus 2 (SARS-CoV-2) on duration of isolation and resource utilization
Source: Infect Control Hosp Epidemiol. 2020 Aug 24:1–3. doi: 10.1017/ice.2020.433 (PMC8245332; doi:10.1017/ice.2020.433)

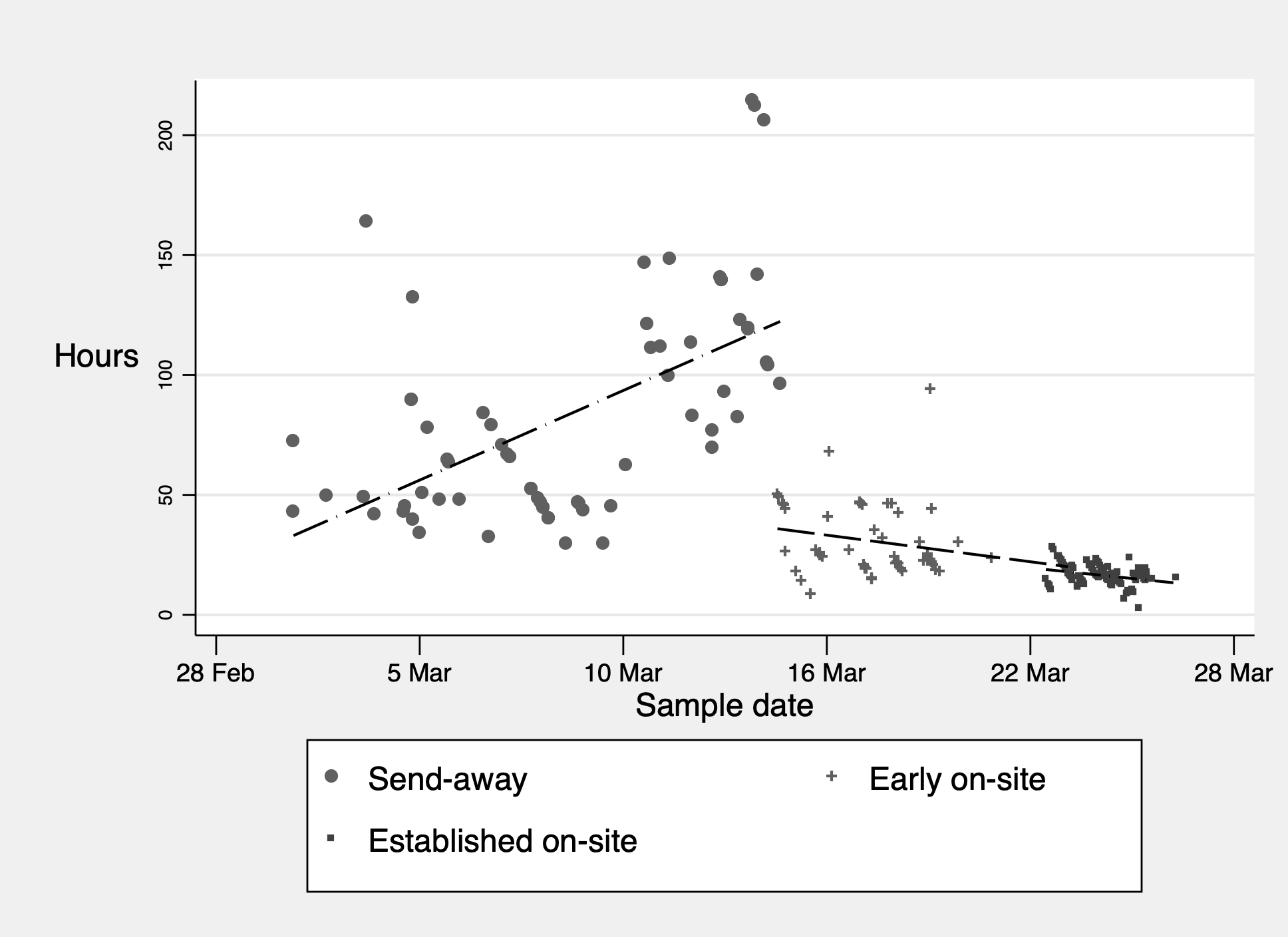

Supplement: Supplementary file 1 [file icesup.zip › S0899823X2000433Xsup001.tif]
